# Efficient Modeling and Simulation of PMUT Arrays in Various Ambients

**DOI:** 10.3390/mi13060962

**Published:** 2022-06-18

**Authors:** Omer M. O. Abdalla, Gianluca Massimino, Alessandro Stuart Savoia, Fabio Quaglia, Alberto Corigliano

**Affiliations:** 1 Department of Civil and Environmental Engineering, Politecnico di Milano, 20133 Milan, Italy; omermohamed.abdalla@polimi.it (O.M.O.A.); gianluca.massimino@polimi.it (G.M.); 2 Dipartimento di Ingegneria Industriale, Elettronica e Meccanica, Università degli Studi Roma Tre, Via della Vasca Navale 84, 00146 Roma, Italy; alessandro.savoia@uniroma3.it; 3 Analog, MEMS & Sensors Group, ST Microelectronics, Via Tolomeo 1, 20010 Cornaredo, Italy; fabio.quaglia@st.com

**Keywords:** piezoelectric micromachined ultrasonic transducers (PMUTs), ultrasound, reduced order modeling (ROM), acoustic–structure interaction, multiphysics modeling

## Abstract

This paper presents a numerical reduced-order modeling (ROM) approach for complex multi-layered arrays of piezoelectric micromachined ultrasonic transducers (PMUTs). The numerical modeling technique adopted to generate an array of PMUTs consisting of a considerable number of transducers allows for a large reduction in computational cost without reducing accuracy. The modeling idea is based on coupling shell elements applied to the PMUT structural layers with 3D-solid elements applied to the piezoelectric layer. A set of eigenfrequency and frequency domain analyses are presented considering a single ROM of a PMUT performing in different ambients and the performing central frequencies are obtained for every considered scenario. A unique arrangement of 228 PMUTs is presented and tested for its ability to transmit and receive acoustic waves. The operating frequency band of the array and the level of interference and cross-talk among different PMUTs in the near field are estimated. Finally, the results from a preliminary experimental test performed to analyze the acoustic abilities of an 8 × 8 array of PMUTs are presented. A corresponding numerical model is created and the obtained results matched the experimental data, leading to a validation of the modeling technique proposed in this work.

## 1. Introduction

Ultrasonic transducers are devices able to generate and detect ultrasonic waves propagating in air, liquid, or solid media. Particularly, piezoelectric micromachined ultrasonic transducers (PMUTs) are devices fabricated on silicon using micro-electro-mechanical systems (MEMS) technologies based on the piezoelectric effect. The applications of piezoelectric materials in smart micro-systems [1], such as PMUTs, are continuously increasing. PMUTs are composite plates that consist of one or more piezoelectric layers sandwiched between two very thin and highly conductive electrode layers that are deposited on top of structural layers. The multi-layered system is suspended on a cavity that provides enough space for the flexural movements of the plates. The active layer in the multi-layered system is typically a thin layer of a piezoelectric film made of lead zirconate titanate (PZT) deposited by sol–gel technique in hat configuration on a structural plate made of silicon [2,3]. The generation of the ultrasonic waves comes from the “inverse effect” of the piezoelectric material, causing a displacement in the membranes by applying an AC voltage signal between the electrodes. The vibrating multi-layered system of PMUTs leads to the generation of an ultrasonic wave that can travel inside the domain of interest such as solids or fluids [4,5,6]. The ultrasonic waves emitted can be used in a wide range of industrial applications such as range finding [7,8], finger-printing recognition [9] and ultrasonic imaging [10,11]. The “direct effect” of piezoelectric material, which refers to the generation of an electrical potential difference under the action of a mechanical strain, is used in PMUTs in energy-harvesting applications to obtain energy from external elements that provide freely mechanical energy [12,13]. In all the mentioned applications, PMUTs are either used as transmitting or as receiving elements of ultrasonic waves [14,15]. To increase the range of transmission and reduce power consumption, there is a need to enhance the sensitivity of the elements. These goals can be reached to combine single PMUTs to create an array.

This work presents the numerical multiphysics modeling and simulation of an array of PMUTs. The main objective is to provide an innovative reduced-order model (ROM) of the array of PMUTs and to investigate its electro-mechanical-acoustic performances. The ROM of the PMUTs is defined by coupling shell and solid elements. The advantage of using shell elements for modeling vibrating plates over solid elements when it comes to meshing and time solutions is self-explanatory. Depending on the size of the model and the capabilities of the hardware, the run-time and the total number of degrees of freedom (DOF) are highly reduced when using shell elements instead of solid elements without compromising the accuracy of the results. Solid elements have no limitations in terms of generating and representing a geometry, while shells are mainly designed to represent structures whose thicknesses are much smaller with respect to the other two dimensions, which is the case in our multi-layered system [16,17]. The model order reduction (MOR) proposed in [6] is based on reducing the dynamic behavior of PMUTs through a linear combination of a few selected piezo-mechanical eigenmodes. On the other hand, the ROM proposed in this work is based on the adoption of first order shear deformation theory (FSDT) shell elements applied to the structural layers and 3D piezoelectric elements applied to the piezoelectric material. This approach does not refer to any pre-selected modes of vibration, therefore it is suitable to capture the dynamic behavior of the transducers involved during the interference phenomena in the complex near-field. The results of the electro-mechanical-acoustic performance of the ROM are compared with a full-order model (FOM), created using only solid elements, which showed a great match indicating the reliability of the ROM technique adopted. The frequency bandwidths of the ROM array in transmission and reception in the near field of an acoustic domain surrounding the PMUTs are evaluated. The presented ROM model for the PMUT array is hence suitable to analyze the frequency-performing bandwidth of the array operating in different ambients. The proposed model is able to evaluate the coupled vibrating response of the transducers affected by the interference phenomena in the complex near-field and the effect of cross-talk among the PMUTs.

This paper is structured as follows. The Section 2 describes the multiphysics finite element modeling and the mechanical properties and behavior of the ROM of a single PMUT operating in a vacuum or coupled with water. Eigenfrequency and frequency domain analyses are performed. The ROM is then validated by comparing the accuracy of results obtained with an FOM using quadratic wedge elements. The computational time of an eigenfrequency analysis on a 5 × 5 PMUTs array is compared between the ROM and the FOM. The main design output is presented in Section 3 where a unique arrangement of multiple reduced-order modeled PMUTs is introduced to generate an array. The array performance is investigated for both the transmission and reception phases. The results obtained from the sensitivity analysis are presented in the form of fractional bandwidth. Section 4 presents a preliminary experimental test carried out to investigate the acoustic capabilities of an 8 × 8 array. A representative model created using PMUTs based on the ROM technique is also presented and the obtained results are validated by comparison with the experimental ones. Finally, Section 5 is devoted to some closing remarks.

## 2. Single PMUT: Full- and Reduced-Order Modeling

**Figure 1:**
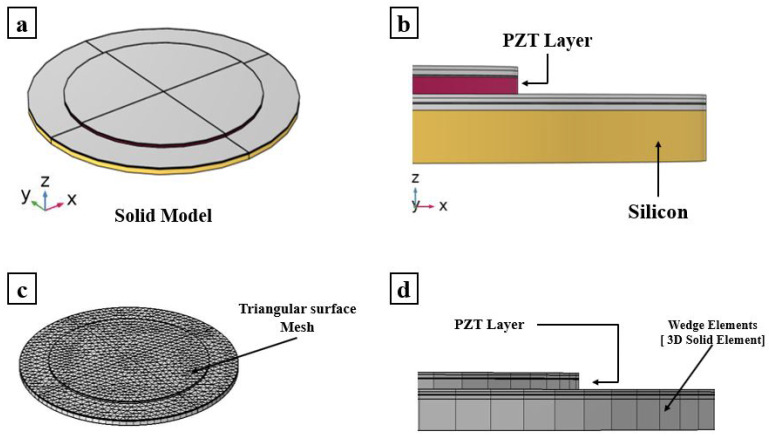
Full-order finite element model of a single PMUT: (**a**) 3D view; (**b**) side view showing the radius of the different layers of the PMUT adopted in the full order numerical model; (**c**) meshing adopted on the external surfaces; and (**d**) side view showing the wedge elements across the PMUT radius.

This section presents the modeling techniques adopted for a single PMUT. The 3D finite element model is created using COMSOL Multiphysics V5.6. The circular diaphragm of the PMUT shown in Figure 1 is a multi-layered thin circular plate which has a structural part made of silicon of a 94 μm radius and a piezoelectric (PZT) active layer coaxial with the diaphragm and a 69 μm radius. The structural silicon layer has a thickness of 4 μm, while the PZT layer has a thickness of 2 μm. The overall thickness of the multi-layered system is 7.445 μm. The PMUT is generally characterized by a high aspect ratio that gives the possibility of having a natural frequency suitable for different applications in a wide range of industrial fields [18].

The ROM technique used to create the PMUT is based on the coupling of 3D solid elements with thin shell structural elements. A linear stress–charge model is used to represent the behavior of the active piezoelectric material.

The “radius-to-thickness” ratio of the multi-layered system is very high. Hence, shell elements are the best candidates to be used to model the different layers of the PMUT. As long as the thicknesses of the layers modeled using shell elements are thin enough, the model ensures highly accurate results.

**Figure 2:**
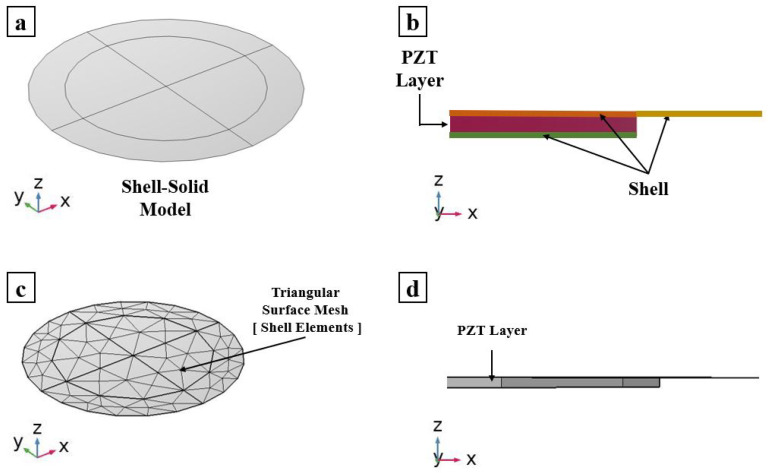
Reduced-order mode of a single PMUT: (**a**) 3D view; (**b**) side view showing radius of the shell and the PZT layer; (**c**) meshing adopted on the external surfaces; and (**d**) side view showing the PMUT radius.

**Figure 3:**
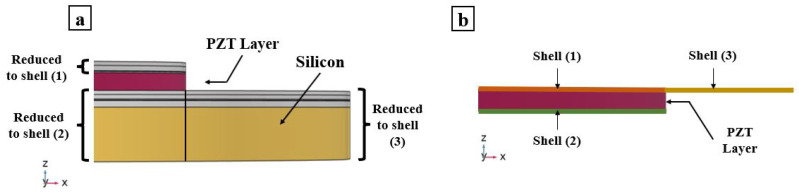
Detailed side view of the PMUT radius showing: the multi-layered configuration of a PMUT radius (**a**); and the reduction of the structural layers of the PMUT into shell elements generating the ROM (**b**).

The multi-layered PMUT is modeled using shell elements apart from the active PZT layer as shown in Figure 2 and Figure 3. The piezoelectric layer is modeled using 3D-solid elements, which are suitable to model the intrinsic 3-dimensional mechanical response of the piezoelectric material. The boundaries and edges on which the solid elements are in contact with shell elements are modeled using boundary conditions that guarantee a good continuity of the displacement field between the shell and solid elements. Shell elements compensate for what they lack in a geometrical sense (as they are 2D) by enriching the information at each node by considering not only translation but also rotations. The shell elements are modeled according to the FSDT. Hence, translations and rotations are considered as DOFs at each node [19]. This displacement field of shell elements is applied to the laminated structural layers of the PMUT. The adopted FEs are quadratic triangular elements. On a shared boundary, the “translation plus rotation” DOFs of the shell nodes need to be connected to the “only translation” DOF of the solid element’s nodes. The kinematic compatibility is thus obtained and enforces the continuity of the displacement field on the solid-shell shared boundaries.

The meshing adopted for the FOM, shown in Figure 1, is entirely composed of quadratic wedge elements. The elements are generated by meshing the surface boundaries of the PMUT using triangular elements of a size equal to $PZT_{r}$/12 in which $PZT_{r}$ represents the radius of the PZT layer. The meshed surface is then used as a reference surface for sweeping through the thickness of each layer of the multi-layered system. Regarding the ROM, the surfaces of the shell elements are meshed with a low element size equal to $PZT_{r}$/2. Only boundaries are meshed for shell elements, as can be seen in Figure 2. For the solid PZT layer, quadratic wedge elements are created by sweeping through the thickness of the triangular surface mesh used in common surfaces shared by the shell elements and the PZT solid layer. A convergence test was conducted on the full-order model of a PMUT to compute the right fundamental frequency and eigenmodes and the frequency response function in immersion presented in this section. In particular, the final adopted discretization applied to the FOM presented in Figure 1 is defined as the one characterized by the smallest element size that gives stable results for any further reduction (finite element convergence). Hence, the proposed ROM mesh, shown in Figure 2, was obtained as the one characterized by the smallest element size that guarantees the convergence to the FOM results.

A set of different models are created to assess the mechanical properties of the ROM for the single PMUT. First, eigenfrequency analysis is performed for the single PMUT in vacuum and in water. A frequency sweep analysis is carried out in the frequency domain and the results of the study are given in terms of the amplitude of the transversal component of the displacement field of the center of the plate.

The analyses described in this section are performed on: (i) the ROM of a PMUT coupling shells and solid elements; and (ii) an FOM of a PMUT created entirely using solid elements. The results obtained from the ROM are compared with FOM to evaluate its accuracy, performance, and the level of computational time reduction in ROM. Great agreement in performance is obtained between the two models.

### 2.1. Analysis on PMUTs in Vacuum

**Figure 4:**
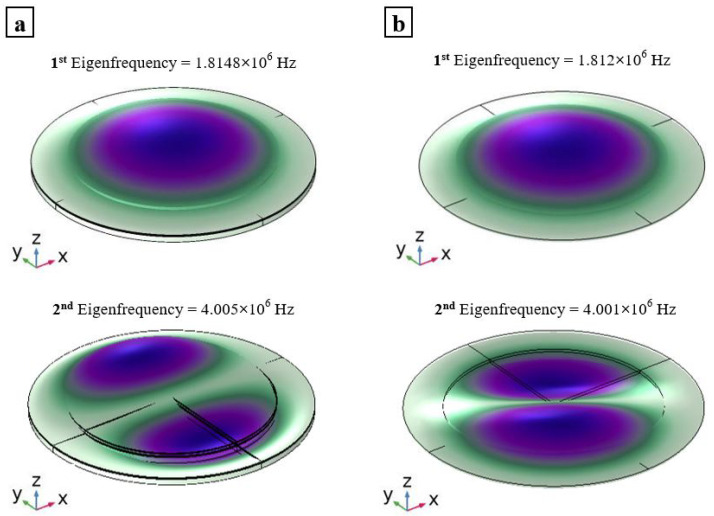
First and second mode shapes and central frequencies in the vacuum of the FOM (**a**); and the ROM (**b**).

A single PMUT in a vacuum is simulated using the mesh presented in the previous section. A total of 156363 DOF are used in the case of the FOM while only 2961 DOF are used for the ROM; hence, a reduction in DOF of almost two orders of magnitude is reported. This represents the simplest case in which the PMUT is not interacting with any other structural element that would affect its natural frequency. The results show very good agreement between the ROM and the FOM. A first mode shape with a central frequency of $f_{r,ROM}$ = 1.812 (MHz) is obtained from the shell-solid coupled model, while the full solid element model gave a first mode-shape with a central frequency of $f_{r,FOM}$ = 1.814 (MHz). Furthermore, higher anti-symmetrical modes are in great agreement, as can be seen in Figure 4. The simulations were run using a workstation with an Intel (R) Xeon (R) CPU E5-1650 V4 @ 3.6 GHz and 64 GB RAM.

**Figure 5:**
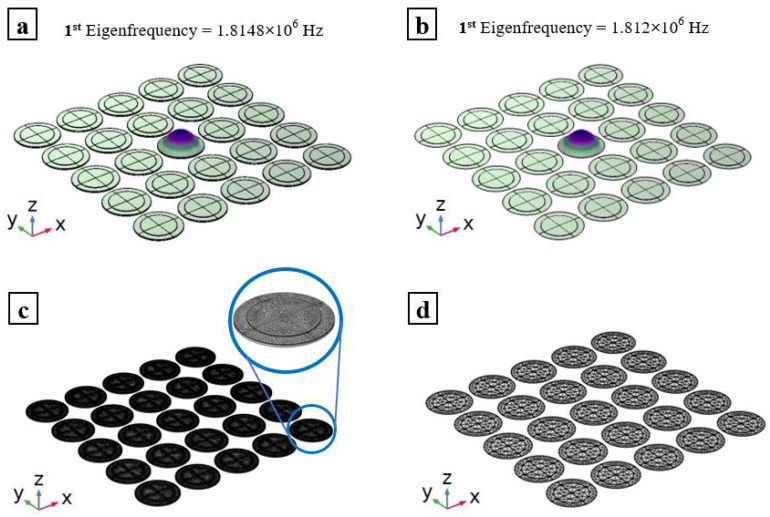
First mode shape and fundamental frequency of the central PMUT in a 5 × 5 PMUTs array in vacuum of the FOM (**a**); and ROM (**b**). Mesh details of FOM of the array (**c**); and ROM of the array (**d**).

The analysis performed on the single PMUT is not computationally demanding and hence it does not show the potential advantages of using the proposed modeling technique, namely the ROM, over the FOM. To better show the advantages of the proposed ROM strategy, the same analysis is carried out on an array of 5 × 5 PMUTs. The mesh adopted to realize the array is built starting from the one used for the single PMUT case presented in Figure 1 and Figure 2 for the FOM and ROM, respectively. A total of 3,909,075 DOF are used for the case of the array created using the FOM technique while only 74,025 DOF are used for the ROM case, maintaining the two-order of magnitude reduction. This study is set to solve for the first mode shape of each PMUT in the array. Using the same computational resources for the single PMUT case, it was found that only 14 s is required to finish the analyses in the case of the ROM, while the FOM needed 27 min and 26 s. The eigenvalue corresponding to the first mode shape of the middle PMUT in the array for both the ROM and the FOM, see Figure 5, is identical to the results obtained for the case of the single PMUT. Hence, it is evident that the proposed ROM technique provides a huge reduction in the computational time, while preserving a great level of accuracy when compared to the FOM. The ROM technique proposed combines a possibility of modeling, with high accuracy, arrays of even much larger dimensions. A high element density PMUTs array would require an extremely large computational time if modeled using the FOM and if coupled with an acoustic domain, the computational time of the multiphysics problem would drastically grow. Hence, the proposed ROM provides an alternative and smart way to strongly reduce the computational burden.

### 2.2. A Single PMUT Immersed in Water

**Figure 6:**
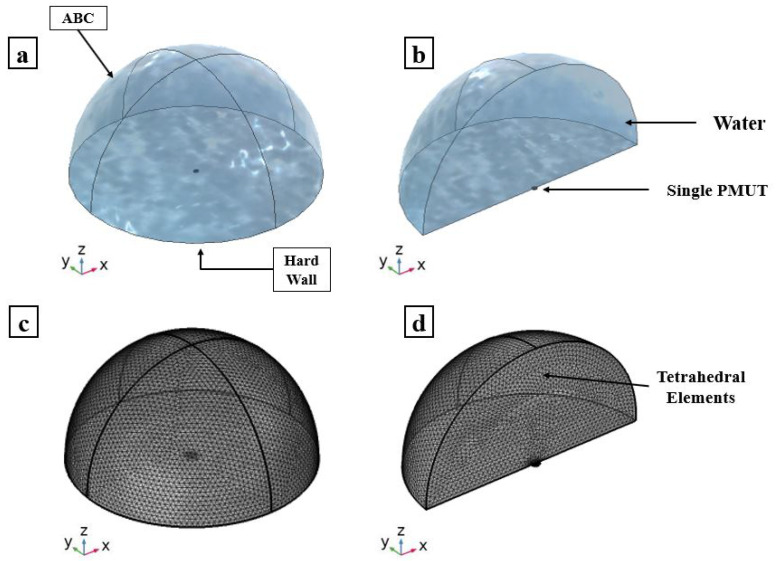
Single PMUT immersed in a water domain: adopted model (**a**); and a plane cut view (**b**). Mesh details of: adopted model (**c**); and a plane cut view (**d**).

**Figure 7:**
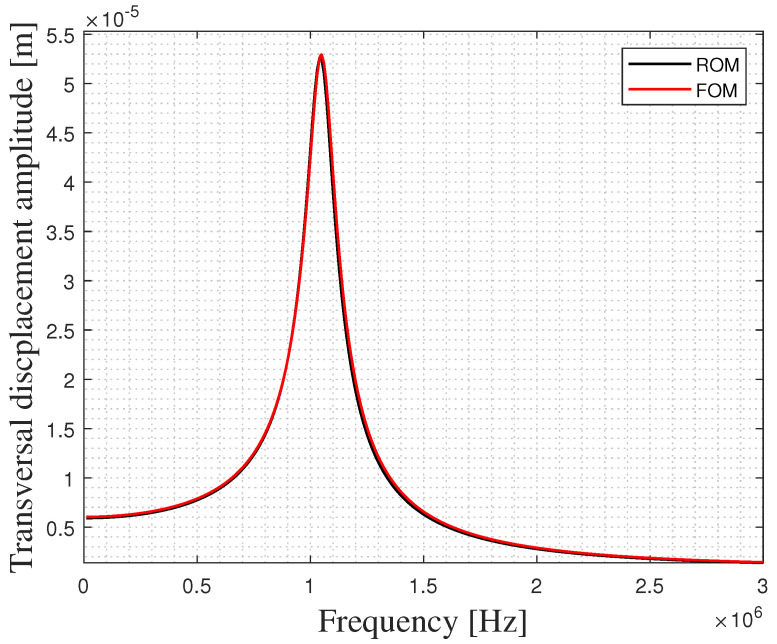
Response spectrum for a single PMUT immersed in water for the ROM and the FOM.

The two models of a single PMUT discussed in the case of vacuum are now coupled with the water domain, as can be seen in Figure 6. The Helmholtz pressure wave equation is solved for in the case of water medium which has a density of 998 (kg/m^3^) and a sound velocity c of 1481.4 (m/s). The water domain is modeled as a hemisphere with a radius of ≈2.5 mm, which is approximately three times the minimum wavelength ($\lambda_{m}$) of the acoustic wave in the water. The value of $\lambda_{m}$ is equal to c/$f_{0}$, in which c and $f_{0}$ are the pressure wave velocity and the resonance frequency of the PMUT in water, respectively.The set of electro-mechanical-acoustic equations governing the multiphysics problem is presented and thoroughly discussed in a previous work of ours [6]. A hard wall boundary condition (HW) is assigned to the lower surface of the hemisphere representing the external highly reflective surface. On the other hand, an absorbing boundary condition (ABC) is assigned to the surfaces around the hemispherical domain to simulate an infinite in-water propagation domain. The acoustic domain is meshed using the quadratic tetrahedral elements of a size equal to $\lambda_{m}$/10 = 8.14 × 10${}^{-4}$ m. The PMUT is meshed as presented in Figure 2. A frequency sweep analysis is performed around the expected central frequency of the PMUT and the results are obtained in terms of the transversal displacement of the center of the PMUT. This analysis is enhanced by adopting the generalized alpha method for time integration. The response spectrum for the two cases is shown in Figure 7. Both cases show a shift in the central frequency towards a lower frequency compared to the case of the vacuum study due to the influence of the fluid mass. A resonance frequency of 1.04 (MHz) is obtained from both the FOM and ROM. The ROM gives an amplitude that is extremely close to the value obtained from the FOM, showing an excellent agreement between the two modeling techniques.

## 3. Array of PMUT: Efficient Modeling via ROM

**Figure 8:**
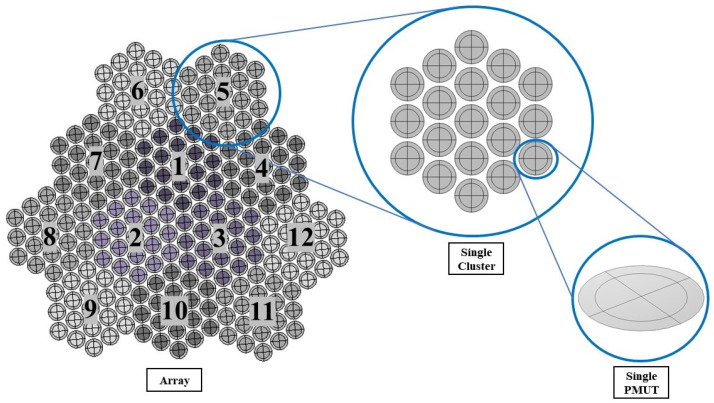
Configuration of the array of PMUTs.

Using the ROM of a single PMUT as a building unit, a unique arrangement of multiple PMUTs is adopted to create the array, as can be seen in Figure 8. A total of 19 single PMUTs arranged in a hexagonal-tiled shape creates a single cluster. All the single PMUTs in the cluster are electrically connected in parallel. The array is made of 12 clusters of PMUTs that are electrically independent, making a total of 228 transducers in an array covering a total surface area of 6.33 × 10${}^{-6}$ $m^{2}$. A pitch of 166 μm is used between the transducers. The total surface of the PZT layers available in this array configuration is equal to 3.41 × 10${}^{-6}$ $m^{2}$. With a thickness of 2 × 10${}^{-6}$ m for each PZT layer, the volume of the active PZT material in the array is equal to 6.82 × 10${}^{-12}$ $m^{3}$.

An array is typically deposited on top of a protective layer that has suitable acoustic properties to enhance the transmission and reception of the acoustic wave. Silicone, which is an elastomeric material, has acoustic properties very similar to water and good adhesive properties making it a good candidate to be used with arrays. Hence, a more realistic model of a system involving the transmission and reception of ultrasonic waves through different media should involve the interaction of the array with the medium through a layer of material such as silicone. Silicone is also found to provide a great match to the acoustic impedance with the human body, making it a good candidate for medical ultrasound probes [20,21]. A 0.5 mm-thick layer with a 2.5 mm radius is used in the transmission and reception of acoustic waves of an immersed array. Three cases are considered in analyzing the transmission in immersion, while two cases are considered for reception, as detailed in Section 3.1 and Section 3.2.

### 3.1. Transmission Phase

**Figure 9:**
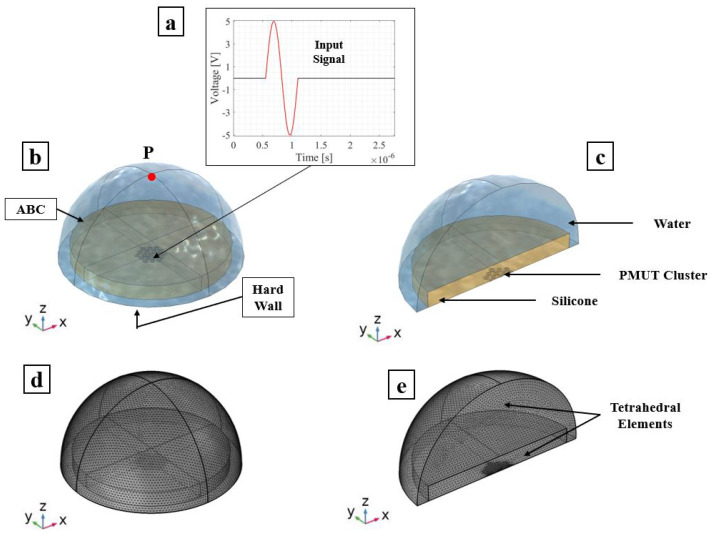
Stand-alone cluster with a silicone matching layer in immersion: PMUT excitation signal (**a**); adopted model (**b**); and plane cut view (**c**). Mesh details of: the adopted model (**d**); and plane cut view (**e**).

**Figure 10:**
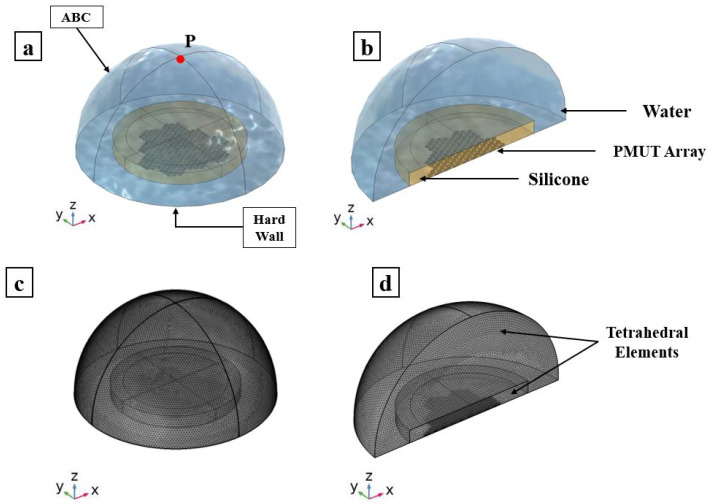
PMUT array in contact with a silicone matching layer in immersion; adopted model (**a**) and plane cut view (**b**); mesh details of adopted model (**c**); and a plane-cut view (**d**).

**Figure 11:**
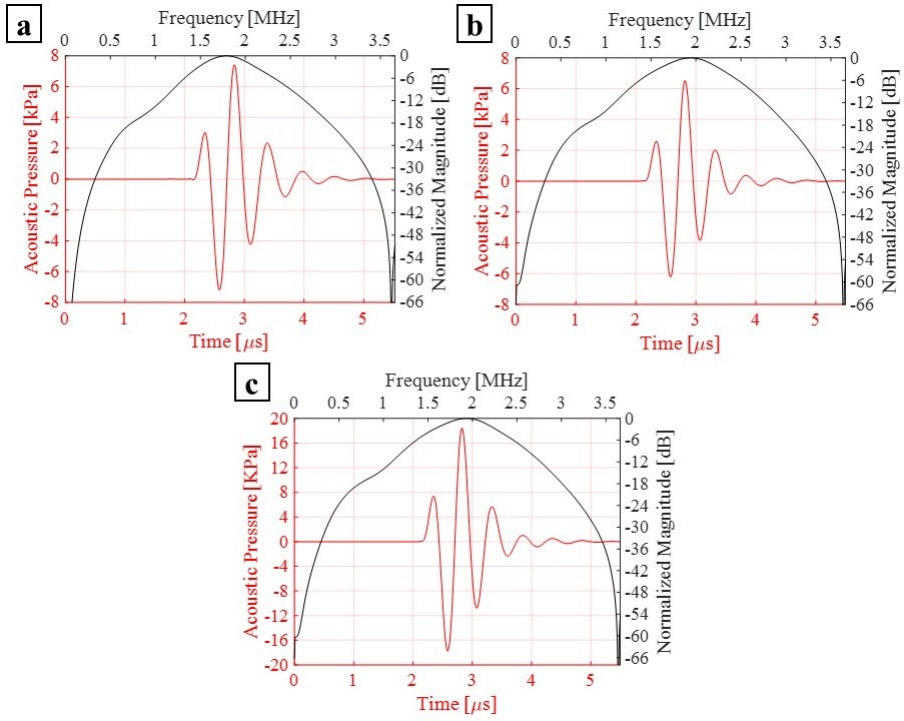
The transmitted pressure, collected at the top point (point P) of the spherical water domain, and the corresponding Fourier transform of the case (i) (**a**); case (ii) (**b**); and case (iii) (**c**).

**Figure 12:**
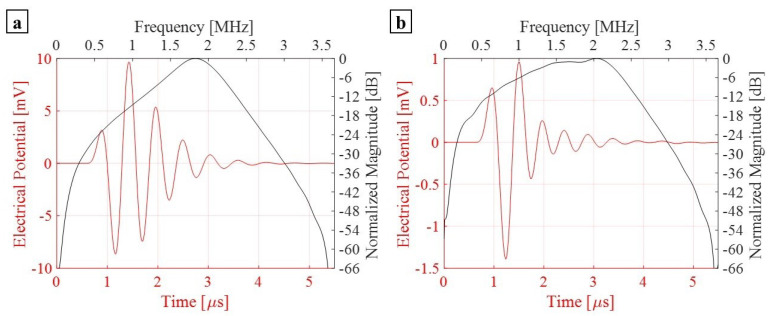
Electrical potential detected from clusters 6 (**a**) and 11 (**b**) of case (ii).

The array of PMUTs is investigated for its performance in the transmission phase at its central frequency. The array is deposited on top of a 0.5 mm-thick cylindrical layer of silicone, which is a rubber-like material acting here as a matching layer immersed in a hemispherical water domain. To simulate a case of immersion, the water domain is considered as infinite with respect to the minimum wavelength ($\lambda_{m}$) of the acoustic wave transmitted from the array into water. This is achieved by assigning an ABC on the external surface of the hemisphere. An acoustic–structural interaction (ASI) condition is enforced on the wet boundaries. In particular, a normal acceleration continuity equation is adopted on the structural–acoustic boundary. The pressure wave equation is solved in the acoustic domain. The electro-mechanical-acoustic problem is solved in the time domain by means of the implicit generalized alpha time integration scheme with a suitable adopted integration time step of 1/(64 × $f_{0}$), in which $f_{0}$ is the central frequency of 1.81 (MHz) at which PMUTs are excited. The meshing adopted for the PMUTs is presented in Figure 2. Both the water and silicone domains are meshed using quadratic tetrahedral elements. The element size is equal to 8.18 × 10${}^{-4}$ m which corresponds to 1/10 of the minimum wavelength ($\lambda_{m}$) of the acoustic wave in water. The numerical scheme and the mesh described are adopted in all the models in this section and in the following one. All the PMUTs of the array are activated using a single cycle sinusoidal input signal with an amplitude of 5 V, with a central frequency of 1.81 (MHz). The acoustic pressure is collected at the top point (point P) of the water domain. A standard Fourier transform analysis is applied on the collected pressure history, and the corresponding response spectrum, normalized with respect to the maximum amplitude, is obtained. The performance of the PMUTs is evaluated on the basis of the fractional bandwidth (FBW) by referring to the two frequencies’ values obtained at −6 dB. Three cases are considered: (i) a stand-alone cluster, see Figure 9; (ii) a single cluster in the center of the array, cluster number 1 shown in Figure 8, is activated as a transmitter while the remaining ones are in a receiving mode; and (iii) clusters 1, 2 and 3, which are active transmitters and the rest are receivers, as can be seen in Figure 10. The first two cases are considered to assess the behavior and the performance of a cluster as a single unit and as a member of an array. The last case is considered as this is one of the preferred configurations in the acoustic experiments. Measuring the bandwidth points at −6 dB level, a fractional bandwidth (FBW) of 56.31% is obtained from the first case, a FBW of 57.94% for the second case and a FBW of 57.25% for the last case. The level of the recorded pressure from case (iii) shows higher intensity when compared to the first two cases as it can be observed from Figure 11. It is worth noting that the pressure intensity transmitted by the stand-alone cluster is slightly higher than that generated from the single active cluster in the array. This is mainly a result of the cross-talk among the different elements in the array. It can be seen from the results presented in Figure 12 that the clusters that are in receiving mode in the second-case scenario generated a certain level of electrical potential, indicating the presence of cross-talk among different PMUT array elements. This acoustic interaction among the different transducers is predictable and occurs at different levels of intensity. It can be seen that cluster 6, which is located directly next to active cluster 1, shows a greater level of interaction, with a 10 (mV) amplitude of electrical potential, than the farther cluster number 11, with an electrical potential amplitude slightly above 1 (mV). Such a phenomenon is undesirable and should be controlled and reduced [22,23]. The cross-talk analysis is possible by means of the presented ROM, which provides a suitable tool to properly assess the level of interaction among the transducer in an array.

### 3.2. Reception Phase

**Figure 13:**
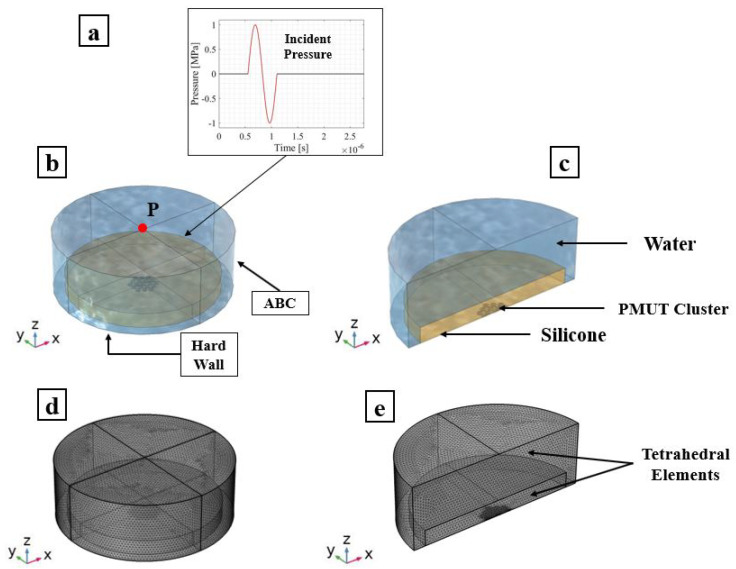
PMUT cluster immersed in a water domain: applied incident pressure (**a**); adopted model (**b**); and plane cut view (**c**). Mesh details of the adopted model (**c**); and plane cut view (**d**,**e**).

**Figure 14:**
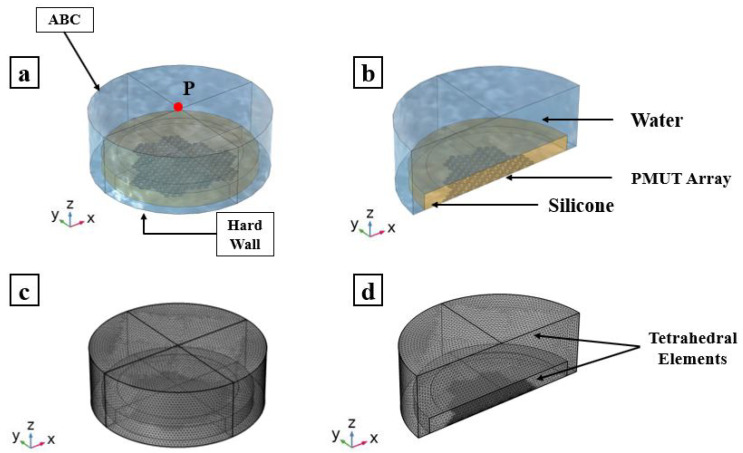
PMUT array immersed in a water domain: adopted model (**a**) and plane cut view (**b**). Mesh details of adopted model (**c**) and plane cut view (**d**).

**Figure 15:**
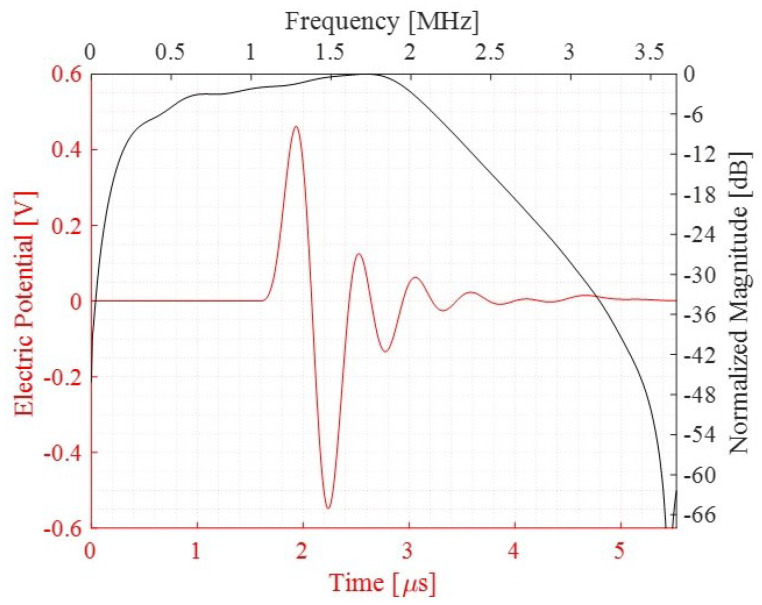
Electrical potential and the corresponding Fourier transform detected from a stand-alone cluster.

**Figure 16:**
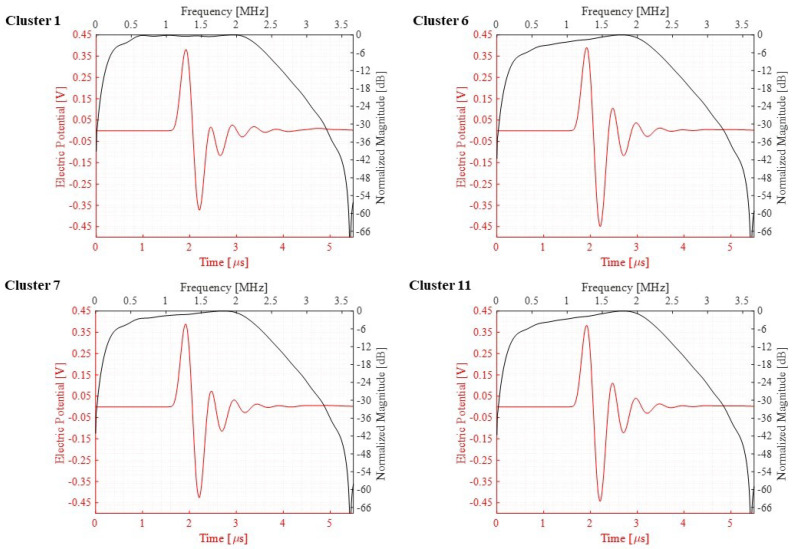
Electrical potential and the corresponding Fourier transform detected from clusters 1, 6, 7, and 11.

The sensitivity of the PMUTs in reception is tested in two scenarios: (i) a standing alone cluster and (ii) an array made of the 12 electrically independent clusters all in receiving mode, as can be seen in Figures Figure 13 and Figure 14. The models in this study also consider the PMUTs to be in contact with the matching layer being immersed in a water domain. The geometry of the water domain is changed in this study to a cylindrical shape to properly model a plane wave incident pressure propagation from its top flat surface. A single cycle sinusoidal pressure history with a central frequency of 1.81 (MHz) and an amplitude of 1 (MPa) is applied to the top surface of the water domain. The voltage is recorded on a single PMUT, the central PMUT, in the first case of the standing alone cluster and in the central PMUTs in each electrically independent cluster in the second case. All the PMUTs in the electrically independent cluster generate the same electric potential at each electrode. Considering the first case, the two frequencies defining the bandwidth, at −6 dB of the voltage gain, are $f_{1}$ = 0.341 (MHz) and $f_{2}$ = 2.149 (MHz), giving a FBW of 133.15%, as can be seen in Figure 15. While Figure 16 presents the voltage gain and the corresponding signal bandwidth from the middle PMUT of Clusters 1, 6, 7 and 11, with reference to the results of the middle cluster, number 1, the two frequencies defining the bandwidth are $f_{1}$ = 0.327 (MHz) and $f_{2}$ = 2.396 (MHz), giving an FBW of 151.96%. The results show a slight difference in the gained voltage among the different clusters. As the incident pressure reaches the PMUT array, the different elements start moving and create pressure as well. Due to the interference of the waves in the near field generated by the vibrating elements, a cross-talk among the transducers will be present and hence there will be a difference in the potential gain from different clusters.

## 4. Experimental Validation

**Figure 17:**
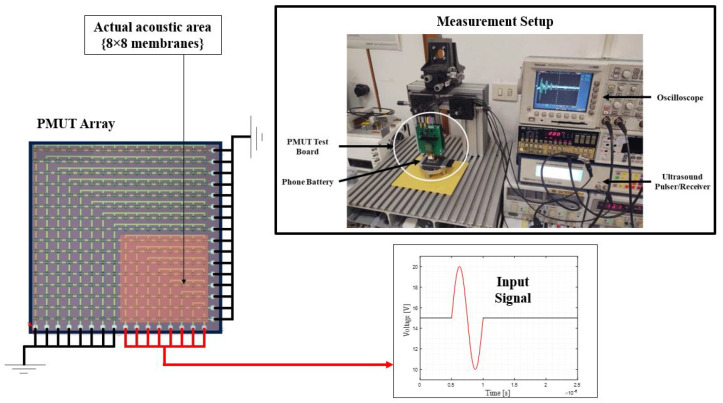
The experimental setup used for the battery and the stainless-steel reflector acoustic test.

This section presents a preliminary experimental campaign carried out to test the acoustic capabilities of an array of PMUTs. The acoustic test is performed on a test object in immersion, a mobile phone battery with an aluminum case. The measurements were conducted using a setup shown in Figure 17 consisting of an ultrasound pulser/receiver, an 8 × 8 array of PMUTs, and an oscilloscope. The setup shows both performances in transmission and reception which is conducted for a battery monitoring application. The measurements took place in a pulse-echo scenario, in which the transducer is used as a transmitter and a receiver. The tested object is separated from the transducer using water as a coupling layer with a thickness of 7.5 mm. Using the same setup, another test is conducted without the presence of the test object but with a stainless-steel perfect reflector. The latter test is conducted to serve as a reference for the former one.

A 16 × 16 membrane fitted in a square configuration defines the transducer used in the acoustic test. Only an 8 × 8 square area is activated in this test. The transmission phase is activated by driving the PMUTs in the array using a single-cycle sinusoidal wave. The input signal is a single-cycle sinusoidal burst characterized by a 10 V peak-to-peak amplitude and a 2 (MHz) frequency. A 15 V DC bias was applied to the PMUT. The experimental setup is shown in Figure 17.

**Figure 18:**
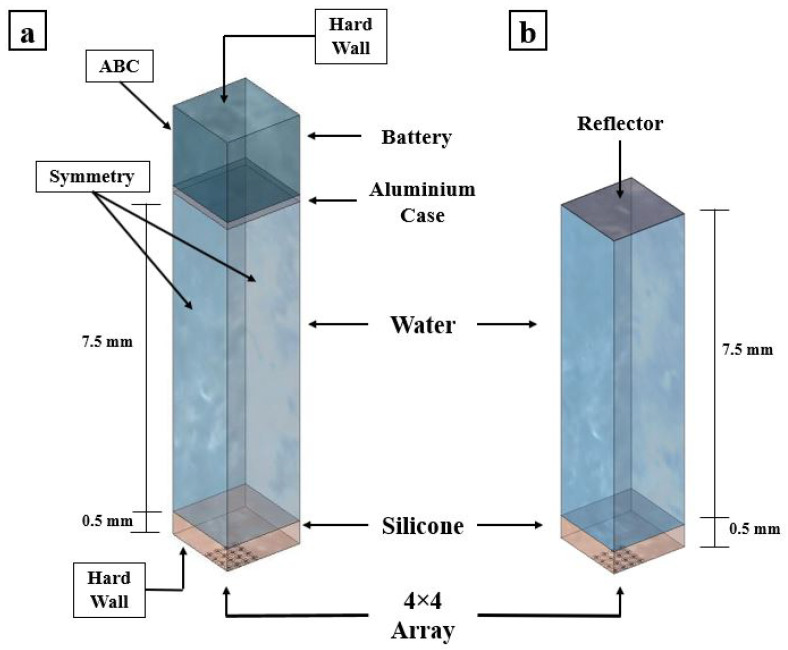
Numerical models representing the cases: mobile phone battery (**a**); and stainless-steel reflector (**b**).

**Figure 19:**
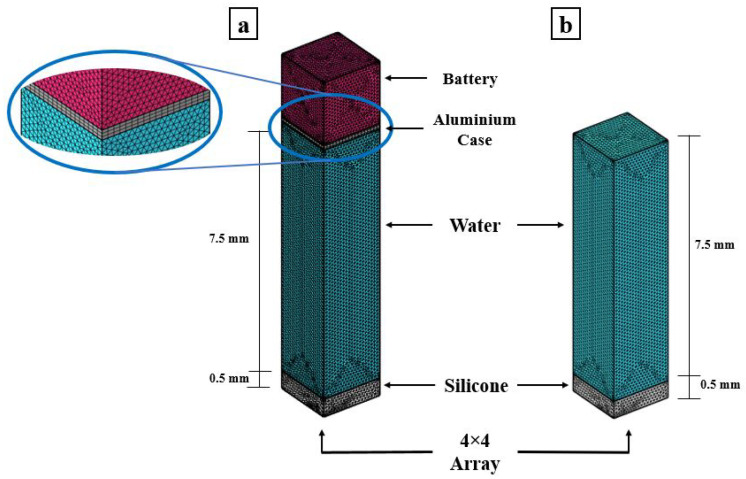
Mesh details of the numerical models representing the cases: mobile phone battery (**a**); and stainless-steel reflector (**b**).

**Figure 20:**
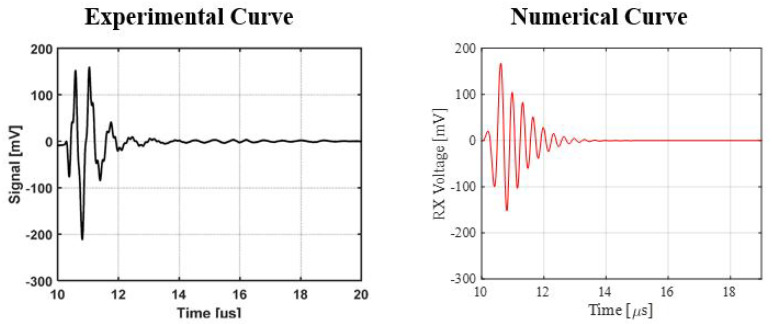
Recorded echo from the reflector for experimental and numerical results.

**Figure 21:**
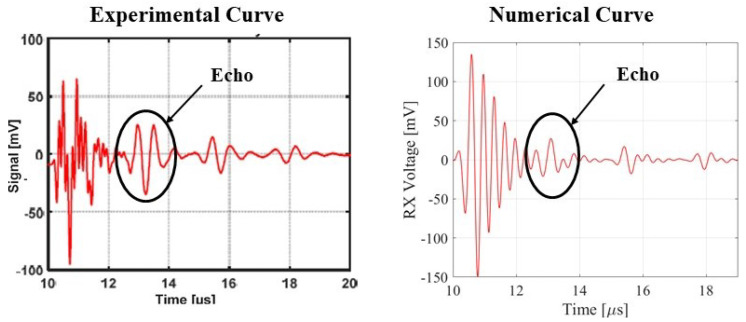
Recorded second echo from the inside of the battery for experimental and numerical results.

**Figure 22:**
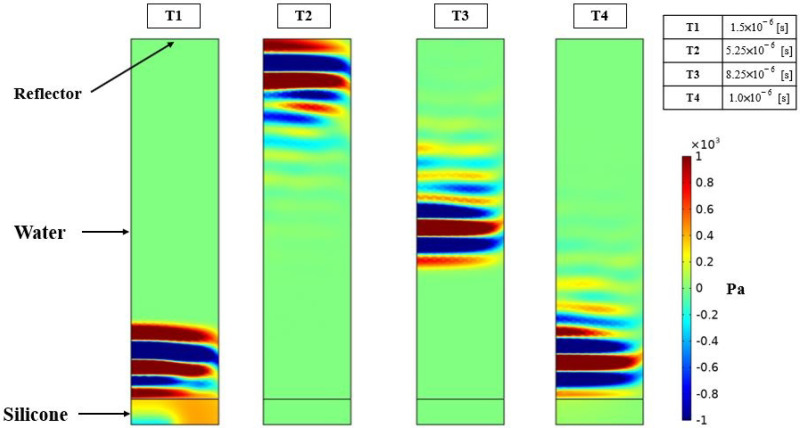
Pressure propagation on a vertical plane of the reflector case at different time instants. (T1) Pressure emitted from the array propagating inside the water domain; (T2) the pressure wave reaches the reflector; (T3) the echo is traveling back towards the array; and (T4) the echo reaches the array which is at a receiving mode.

**Figure 23:**
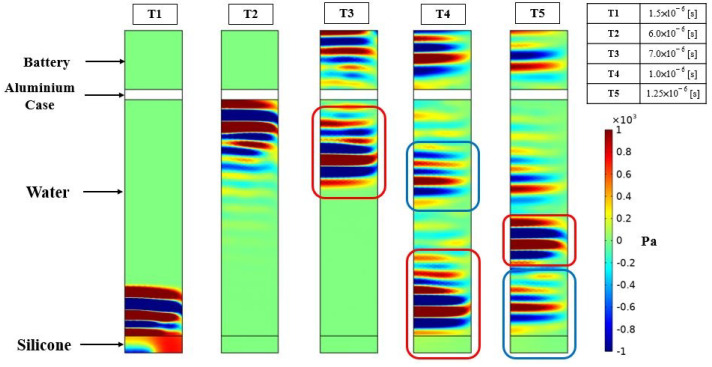
Pressure propagation on a vertical plane of the battery case at different time instants. (T1) Pressure emitted from the array propagating inside the water domain; (T2) the pressure wave reaches the aluminum case of the battery; (T3) part of the pressure wave is transmitted inside the battery and another part is reflected by the aluminum case, the first echo highlighted in red box is (T4) the instant when the first echo reaches the array. A second echo coming from the inside of the battery is evident, highlighted in a blue box; and (T5) the instant at which the echo from inside of battery reaches the array.

A numerical FEM is generated to simulate the two testing cases. The array is created using the proposed ROM of PMUTs. Since the configuration of the array is square in shape, modeling the two cases is less computationally expensive if the symmetry property of the setup is utilized. Hence, only a 4 × 4 array of PMUTs is modeled to represent the experimental setups, as shown in Figure 18. The meshing adopted for the PMUTs is the one presented in Figure 2. Both the battery and the water domain are meshed using quadratic tetrahedral elements of a size equal to 1/10 which is the minimum wavelength ($\lambda_{m}$) of acoustic wave in water, equal to 7.4 × 10${}^{-4}$ m. The value of $\lambda_{m}$ is obtained in reference to the central frequency $f_{0}$ = 2 (MHz), at which the array is excited, and to the pressure wave velocity c = 1481.4 (m/s) in water. The aluminum case is meshed using quadratic wedge elements, as can be seen in Figure 19. The integration scheme and time step are the ones adopted in the previous examples. Figure 20 shows the recorded echo from the reflector for the numerical and experimental test. The results of the recorded second echo from the inside of the battery obtained from the numerical model show great agreement with the experimental test for the case of the battery. Figure 21 shows a recorded second echo of 50 (mV) peak-to-peak obtained due to the reflection of the acoustic wave from the battery. Both the recorded voltage amplitude and time of flight of the echo from the two cases are matching. The echoes presented in Figure 20 and Figure 21 can be seen in the pressure propagation maps presented in Figure 22 and Figure 23.

## 5. Closing Remarks

This paper proposes a complete set of modeling techniques to create an ROM of large arrays of PMUTs with a high level of accuracy. The strategy adopted to create the ROM is based on coupling solid elements with shell elements to create the multi-layered system of PMUTs. The combination of them and the application to the modeling and simulation of the PMUTs large array outlines the novelty of this work. Thee validation of the functionality and accuracy of the ROM are investigated by considering PMUTs operating in different scenarios, in vacuum and in immersion.

A set of eigenfrequency and frequency domain analyses are carried out on both the ROM and the FOM, referring only to a single PMUT, considering different scenarios. Results are obtained in terms of the operating central frequencies and the corresponding transversal displacement of the central point of PMUTs. The results show very good agreement between the two modeling techniques, hence confirming the validity of the ROM technique adopted. To compare the computational time reduction, an eigenfrequency analysis, solving for the first eigenvalue corresponding to the first mode shape of the PMUT, was carried out on a 5 × 5 array. The FOM array required 27 min and 26 s to run the simulation while the ROM took only 14 s. Hence, the ROM provides a great reduction in the DOF and the computational time needed for the analysis of the complex system of PMUTs without reducing the accuracy of the system.

Once the ROM of a PMUT is validated, a large number of PMUTs are modeled in a special array configuration. The proposed array is tested for its abilities in both the transmission and reception phases. The proposed ROM of an array showed an FBW of 57.25% in transmission and an FBW of 151.96%. The advantages of using the proposed approach stand on the possibility of very fast and accurate evaluations of the PMUTs array performances including the effect of the multiple interactions among the transducers occurring in the near field. Therefore, extremely accurate and useful cross-talk estimations are possible. Hence, the adopted reduced approach represents an excellent alternative to the full-order model that would require an extremely large computational time.

A preliminary experimental test was carried out to study the acoustic capabilities of an 8 × 8 PMUTs array. The setup was created for battery-monitoring applications. The test considered two cases of a pulse-echo scenario in immersion: (i) a mobile phone battery; and (ii) a stainless steel reflector. Results are collected from the array in terms of recorded voltage that represents the echo of the reflected waves. Numerical models using the ROM of PMUTs are developed for the two cases and the recorded voltage in the array showed high agreement in the magnitude of the recorded voltage and the time at which the echo is recorded. Future work will concern deeper analysis of the cross-talk among the transducers. The performance of the proposed ROM not only on fluid domains but also on solids like in the structural health monitoring applications will be also tested.

## Data Availability

Not applicable.

## Abbreviations

The following abbreviations are used in this manuscript:

ABC	Absorbing Boundary Condition
ASI	Acoustic–Structural Interaction
CPU	Central Processing Unit
DOF	Degrees of Freedom
FBW	Fractional Bandwidth
FOM	Full-Order Model
FSDT	First Order Shear Deformation Theory
HW	Hard Wall Boundary Condition
MEMS	Micro-Electro-Mechanical Systems
MOR	Model Order Reduction
MUTs	Micromachined Ultrasonic Transducers
PMUTs	Piezoelectric Micromachined Ultrasonic Transducers
RAM	Random-Access Memory
ROM	Reduced-Order Modeling
